# Clinical characteristics and genotype-phenotype correlation analysis of familial Alzheimer’s disease patients with pathogenic/likely pathogenic amyloid protein precursor mutations

**DOI:** 10.3389/fnagi.2022.1013295

**Published:** 2022-10-14

**Authors:** Yingzi Liu, Xuewen Xiao, Hui Liu, Xinxin Liao, Yafang Zhou, Ling Weng, Lu Zhou, Xixi Liu, Xiang-yun Bi, Tianyan Xu, Yuan Zhu, Qijie Yang, Sizhe Zhang, Xiaoli Hao, Weiwei Zhang, Junling Wang, Bin Jiao, Lu Shen

**Affiliations:** ^1^Department of Neurology, Xiangya Hospital, Central South University, Changsha, China; ^2^National Clinical Research Center for Geriatric Disorders, Central South University, Changsha, China; ^3^Department of Geriatrics, Xiangya Hospital, Central South University, Changsha, China; ^4^Engineering Research Center of Hunan Province in Cognitive Impairment Disorders, Central South University, Changsha, China; ^5^Hunan International Scientific and Technological Cooperation Base of Neurodegenerative and Neurogenetic Diseases, Changsha, China; ^6^Key Laboratory of Hunan Province in Neurodegenerative Disorders, Central South University, Changsha, China; ^7^Department of Radiology, Xiangya Hospital, Central South University, Changsha, China; ^8^Key Laboratory of Organ Injury, Aging and Regenerative Medicine of Hunan Province, Changsha, China

**Keywords:** Alzheimer’s disease, APP mutations, clinical characteristics, genotype-phenotypic, non-dementia symptoms, neurological symptoms

## Abstract

Alzheimer’s disease (AD) is a progressive neurodegenerative disease associated with aging, environmental, and genetic factors. Amyloid protein precursor (*APP*) is a known pathogenic gene for familial Alzheimer’s disease (FAD), and now more than 70 *APP* mutations have been reported, but the genotype-phenotype correlation remains unclear. In this study, we collected clinical data from patients carrying APP mutations defined as pathogenic/likely pathogenic according to the American college of medical genetics and genomics (ACMG) guidelines. Then, we reanalyzed the clinical characteristics and identified genotype-phenotype correlations in APP mutations. Our results indicated that the clinical phenotypes of *APP* mutations are generally consistent with typical AD despite the fact that they show more non-demented symptoms and neurological symptoms. We also performed genotype-phenotype analysis according to the difference in APP processing caused by the mutations, and we found that there were indeed differences in onset age, behavioral and psychological disorders of dementia (BPSD) and myoclonus.

## Introduction

Alzheimer’s disease (AD) is a progressive neurodegenerative disease characterized by progressive memory loss and cognitive decline (Sperling et al., 2011). According to the World Alzheimer Report 2018, 50 million people were living with dementia worldwide in 2018 (Alzheimer’s Disease International Consortium, 2018), and the number will more than triple to 152 million by 2050. The typical pathological feature of AD is extracellular deposits of amyloid-β (Aβ) plaques and intracellular neurofibrillary tangles (Braak and Braak, 1996). Although the pathological changes of AD are relatively clear, the exact pathogenesis of this disease is still uncertain (Scheltens et al., 2016). AD is widely believed to be associated with aging, environmental and genetic factors (Farrer et al., 1997; Flicker, 2010; Barnes and Yaffe, 2011).

Alzheimer’s disease can be divided into familial Alzheimer’s disease (FAD) and sporadic Alzheimer’s disease (SAD), depending on whether there is a positive family history. Mutations in the amyloid protein precursor (*APP*), presenilin-1 (*PSEN1*), and presenilin-2 (*PSEN2*) genes can cause FAD. These three causative genes explained 5–10% of FAD (Loy et al., 2014; Cacace et al., 2016), and over 200 mutations in these genes have been described so far (Alzforum mutation database).^1^

Amyloid protein precursor mutations are the second most common pathogenic gene for AD, with an estimated mutation frequency of 1% (Cacace et al., 2016; Hinz and Geschwind, 2017). *APP* gene is positioned on chromosome 21q21.2–21q21.3 and has several different isoforms, of which the three most common isoforms are the 695 amino acid form, the 751, and the 770 amino acid forms (Bayer et al., 1999). APP695 is mainly produced by neurons, while APP751 and APP770 are primarily expressed on peripheral cells and platelets (Hardy, 1997; Guerreiro et al., 2012). All three isoforms consist of a single membrane-spanning domain, a large extracellular glycosylated *N*-terminus, and a shorter cytoplasmic *C*-terminus (Muresan and Ladescu Muresan, 2015) and can generate Aβ after sequential sequencing cleavages by β-secretase and γ-secretase (Nunan and Small, 2000). More than 70 *APP* mutations have been reported possibly associated with FAD since the first mutation V717I was discovered (Goate et al., 1991), and most of the mutations were found to increase the production of Aβ or alter the ratio of Aβ_42_/Aβ_40_ (Citron et al., 1992; De Jonghe et al., 2001; Kirkitadze et al., 2001; Nilsberth et al., 2001). Despite one research summarizing the *APP* missense mutations and their impacts on APP Processing (Theuns et al., 2006), the research on the genotype-phenotype of *APP* mutation is limited (Lindquist et al., 2009; Ryan et al., 2016; Jiang et al., 2019). Only a part of *APP* mutations targeted at specific populations was described, and there was no systematic summary of all *APP* mutations. However, to study the pathogenesis of AD better, it is imperative to fully understand its clinical characteristics and the correlations between genotype and phenotype.

In a previous study, we had systematically re-evaluated *APP*, *PSEN1*, and *PSEN2* mutations according to the American college of medical genetics and genomics and the association for molecular pathology (ACMG-AMP) guidelines (Xiao et al., 2021). In this study, we collected detailed clinical data from FAD with *APP* mutations that were re-evaluated as pathogenic/likely pathogenic based on previous research. Then we reanalyzed the clinical characteristics and identified the genotype-phenotype correlations in AD caused by pathogenic/likely pathogenic *APP* mutations.

## Materials and methods

### Data sources and selection

We conducted a literature search using databases from the Alzforum mutation database (see text footnote 1) and PubMed with the keywords “APP” and “Alzheimer’s disease.” All the articles we included in this research described either clinical characteristics and/or neuropathological features. Almost all cases in these articles were diagnosed with AD according to the National Institute of Neurological and Communicative Disorders and Stroke-Alzheimer’s Disease and Related Disorders Association (NINCDS-ADRDA) (McKhann et al., 1984) criteria or the National Institute on Aging-Alzheimer’s Association (NIA-AA) (McKhann et al., 2011). *APP* mutations defined as variant of uncertain clinical significance (VUS) and benign/likely benign were excluded. Asymptomatic individuals and mild cognitive impairment (MCI) were also ruled out from the study.

### Data extraction

The information collected directly from relevant manuscripts was related to the demographic data (origin, gender), age at onset (AAO), onset symptom, clinical feature, disease duration (calculated only for deceased patients), *APOE* allele, neuroimaging, electroencephalography (EEG), cerebrospinal fluid (CSF) biomarkers and the neuropathology for individually affected patients. Data were also extracted when the exact AAO and disease duration were unavailable in the article and the mean AAO, range, and number of patients.

### Statistical analysis

The statistical analyses have been performed using the ANOVA test for continuous variables, chi-square for categorical variables, and correlation analysis for clinical phenotypes. All data were tested for normality and homogeneity of variance using the Shapiro-Wilk and Levene variance equality tests. All data were analyzed with IBM SPSS Statistics (version 23.0) and visualized using Prism 8 (GraphPad). Tests were considered statistically significant for *P* < 0.05.

## Results

### The overall clinical characteristics of pathogenic/likely pathogenic amyloid protein precursor mutations

**FIGURE 1:**
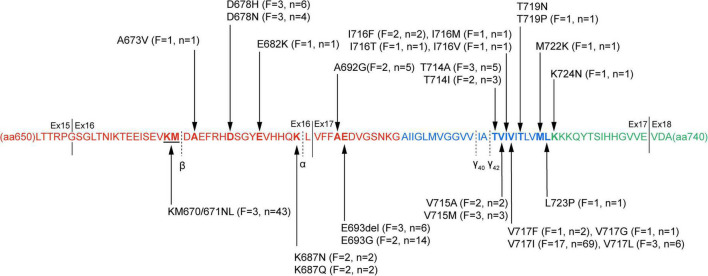
The pathogenic/likely pathogenic mutations in amyloid protein precursor (APP) protein. APP protein sequence from amino acid residue 650–740 is presented. The sequence in red depicts the extracellular domain, the transmembrane domain blue, and the intracellular domain green. Black arrow markers indicate pathogenic/likely pathogenic mutation sites, and the specific information on the mutation is described. The numbers of families and individuals affected are also shown in the figure. The cleavage sites of α-, β-, and γ secretases are marked with black dotted lines, and solid lines separate the different exons. *F*, the number of families affected in the mutations; *n*, the number of affected individuals; Ex, exon.

A total of 31 *APP* mutations were re-evaluated as pathogenic/likely pathogenic based on the previous study, among which 28 mutations related to AD were included in this study, including 26 missense mutations, one double codon mutation, and only one mutation had a single base deletion. All the mutations were located in exon 16 (*n* = 7) and exon 17 (*n* = 21), near the splice site of α secretase, β secretase, and γ secretase. Several other mutations were near α, β, or γ secretase cleavage sites such as V669L or in the other region of *APP* than exon 16–17, but their pathogenic nature was questioned according to ACMG-AMP guidelines. Overall, 63 pedigrees exhibiting *APP* mutations were reported in this research, accounting for 180 affected subjects. Figure 1 shows the locations of *APP* gene mutations, the number of families and individuals affected by the mutations. The most frequently reported mutations were KM670/671NL (*n* = 43, near β cleavage site) and V717I (*n* = 69, near γ_42_ cleavage site).

The clinical characteristics of *APP* gene mutations are summarized in Supplementary Table 1. The overall mean AAO in *APP* mutations was 50.7 years, ranging from 31 to 65 years. Gender information was available in 130 patients, including 55 male patients and 75 female patients (the ratio was 1:1.36). 74 cases reported the onset symptoms, and 170 described the clinical manifestations. Most cases had typical characteristics of memory loss (90.59%, 154/170), some patients also had non-amnestic clinical features including disorientation (39.41%, 67/170), visuospatial disorder (12.94%, 22/170) language impairment (27.65%, 47/170), apraxia/agnosia (49.41%, 84/170), dyscalculia (8.24%, 14/170), behavioral and psychological disorders of dementia (BPSD) (53.53%, 91/170). Some patients with *APP* mutations (e.g., A673V, A692G, and E693del) described neurological symptoms such as extrapyramidal symptoms (EPS), myoclonus, seizures, spastic paraplegia, and ataxia. And for those individuals who reported *APOE* allele, 70.49% (43/61) were *APOE* ε4 negative, 21.31% (13/61) had one *APOE* ε4, and 8.20% (5/61) had two copies of *APOE* ε4.

### The age at onset and disease duration

Age at onset data were available in 123 subjects, in which only 9 (7.32%) patients had AAO <40 years old, and 12 (9.76%) had AAO >60 years old. The AAO of most patients was between 40 and 60 years old. Considering the different mutations, the lowest mean AAO was observed among the A673V and I716F mutations (36.0 and 38.0 years, respectively), while the highest was in the I716M mutation (64 years). We also noted that the AAO in some mutations (e.g., K687N and A692G) were roughly the same, while some mutations (e.g., D678N) showed wide variation. Age at onset could be significantly different among the affected individuals, even within the same family. In our research, the *APOE* allele did not affect the overall mean AAO of *APP* mutations, whereas, in the V717I mutation, carriers of the ε4 allele had an earlier AAO (*p* = 0.005).

The course of AD with *APP* mutations was slow. The mean duration of the disease ranged from 3 to 18 years, with an average of 8.7 years. A faster disease duration with a mean duration of 4 years was observed in the T714I carriers, while the patients with the V717I and V715M mutation experienced a more extended period of the disease (mean duration of 9.9 and 10.0 years, respectively). Similar to the AAO, the course of AD was found to vary widely in the same mutations, even within the same pedigrees.

### The first symptoms and clinical presentation

Most of the patients reported amnesia as the first symptom (82.43%, 61/74), which was throughout almost all subjects and gradually aggravated. Disorientation, BPSD, or functional executive function impairment could be the initial symptoms in some individuals, accounting for 12.16% (9/74) in total. Besides, some patients (5.41%, 4/74) could also exhibit headache, vertigo, and other non-dementia symptoms as the first clinical manifestation. In comparison, no cases reported language impairment and dyscalculia at the onset.

With the progression of dementia, BPSD became the second most common clinical feature after amnesia. BPSD were classified into three subsyndromes, including psychotic syndrome (hallucinations and/or delusions), affective syndrome (agitation and/or depression and/or anxiety and/or irritability), and behavior syndrome (euphoria and/or apathy and/or disinhibition and/or aberrant motor behavior) (Garre-Olmo et al., 2010). Among the above three subsyndromes, the affective syndrome has the highest frequency (75.0%, 63/84) in carriers who reported BPSD, with depression and anxiety being the most common. Hallucinations and delusions of psychiatric symptoms were also frequent in *APP* mutations; Supplementary Table 1). Apraxia/agnosia was also a frequent clinical manifestation in AD, occurring in nearly half of the cases. Over 25% of subjects had disorientation and language impairment, while dyscalculia was rarely reported in dementia cases. We also found that individuals in the same pedigrees tended to be impaired in similar cognitive domains.

Neurological symptoms were concentrated in some of these *APP* mutations, with more than half of the cases clustered in the V717I mutation. In contrast, the A673V and D678N mutations only showed neurological symptoms in one and two patients, respectively. EPS was the most common atypical neurological feature in pathogenic/likely pathogenic *APP* mutations. Patients reported with EPS have at least one clinical manifestation of bradykinesia, rigidity, dystonia, stooped posture, and shuffling gait. Some patients also had other neurological symptoms besides EPS, such as myoclonus, seizures, spastic paraplegia, and ataxia. Pathological reflexes and frontal release signs were shown in *APP* mutations as well. Myoclonus, seizures, spastic paraplegia, and ataxia occur similarly in our research. However, myoclonus and seizures appeared in almost all mutations that reported neurological symptoms, whereas spastic paraplegia was only present in A673V, E693del, T714A, and V717I, ataxia only in E693del, I716F, and V717I. In addition to typical clinical features and neurological symptoms, a tiny percentage of individuals had headaches, vertigo, sleep disturbance, and other non-dementia symptoms (Supplementary Table 1).

### The diagnostic findings

The neuroimaging, CSF, EEG, and neuropathology results were collected in Table 1. Most cases undergoing CT/MRI examination showed diffuse cerebral atrophy or a local involvement of parietal and temporal lobes or hippocampal region, accompanying with or without white matter lesion and other signs. All patients who received positron emission tomography (PET) and/or single-photon emission computed tomography (SPECT) were confirmed to have the following pattern, a temporal and parietal hypoperfusion/hypometabolism at first, then a progressive involvement in the frontal and occipital regions, even the precuneus, and finally the entire cerebral. However, two patients with E693del showed no difference in ^11^C-labeled Pittsburgh compound B (^11^C-PIB) imaging compared with non-demented people, and one harboring E693G mutation had no abnormal results in SPECT.

**TABLE 1:** Neuroimaging, electroencephalography, cerebrospinal fluid (CSF) analysis, and neuropathology findings in pathogenic/likely pathogenic amyloid protein precursor (*APP*) mutations.

Exon	Mutation	CT/MRI	PET/SPECT	EEG	CSF	Neuropathology	References
16	KM670/ 671NL	–	–	–	–	*n* = 3 AP, NFTs	Mullan et al., 1992; Bogdanovic et al., 2002
	A673V	*n* = 1 diffuse atrophy white mater lesion	–	*n* = 1 diffuse slow waves	*n* = 1 Aβ_42_↓	–	Di Fede et al., 2009
	D678H	*n* = 3 diffuse atrophy *n* = 2 diffuse atrophy white mater lesion *n* = 1 diffuse atrophy amyloid angiopathy	*n* = 5 ^18^F-AV-45 PET F-T-P-Pre C-O hypometabolism *n* = 1 SPECT P-T hypoperfusion	*n* = 1 diffuse slow waves	–	–	Chen et al., 2012; Huang et al., 2019
	D678N	*n* = 3 diffuse atrophy *n* = 1 P-T atrophy	*n* = 1 SPECT P-T hypoperfusion	–	*n* = 1 Aβ_42_↓, T-Tau↑, P-Tau↑	–	Wakutani et al., 2004; Han et al., 2020; Mao et al., 2021
	E682K	*n* = 1 hippocampal atrophy	*n* = 1 ^11^C-PIB hypometabolism	–	*n* = 1 Aβ_42_↓, T-Tau↑, P-Tau↑	-	Zhou et al., 2011
	K687N	*n* = 1 diffuse atrophy *n* = 1 diffuse atrophy white mater lesion	–	–	*n* = 1 Aβ_42_↓, T-Tau↑, P-Tau↑	–	Kaden et al., 2012; Xu et al., 2018
	K687Q	*n* = 1 diffuse atrophy	–	–	–	–	Jiang et al., 2019
17	A692G	*n* = 1 local cerebral hemorrhage *n* = 1 diffuse atrophy white mater lesion	–	*n* = 1 Alpha rhythm, disturbance of frontal activity	–	*n* = 3 AP, NFTs	Cras et al., 1998; Brooks et al., 2004
	E693del	*n* = 4 diffuse atrophy	*n* = 1 ^18^F-FDG hypometabolism *n* = 2 ^11^C-PIB normal *n* = 1 ^11^C-PIB T-P-O slight hypometabolism *n* = 1 ^11^C-PIB F-T-P slight hypometabolism	–	*n* = 2 Aβ_42_↓, T-Tau↑, P-Tau↑ *n* = 1 T-Tau -, P-Tau -	–	Tomiyama et al., 2008; Shimada et al., 2011, 2020; Kutoku et al., 2015
	E693G	*n* = 3 diffuse atrophy *n* = 4 white mater lesion	*n* = 1 SPECT normal *n* = 4 SPECT P hypoperfusion *n* = 1 SPECT P-T hypoperfusion	–	–	*n* = 4 AP, NFTs	Basun et al., 2008; Kalimo et al., 2013
	T714A	*n* = 2 diffuse atrophy *n* = 1 white mater lesion	*n* = 1 ^18^F-FDG hypometabolism SPECT P-T hypoperfusion	–	*n* = 1 Aβ_42_↓, T-Tau↑, P-Tau↑	–	Pasalar et al., 2002; Zekanowski et al., 2003; Lindquist et al., 2008, 2009
	T714I	*n* = 2 diffuse atrophy	–	–	*n* = 1 Aβ_42_↓, T-Tau↑	*n* = 1 AP, NFTs	Kumar-Singh et al., 2000; Edwards-Lee et al., 2005
	V715A	–	*n* = 1 SPECT P-O hypoperfusion	–	–	–	Cruts et al., 2003; Zekanowski et al., 2003
	V715M	*n* = 1 diffuse atrophy *n* = 1 temporal atrophy	–	*n* = 1 diffuse slow waves	–	*n* = 1 AP, NFTs	Ancolio et al., 1999; Park et al., 2008; Nan et al., 2014
	I716F	*n* = 1 temporal atrophy *n* = 1 P-F atrophy	*n* = 1 SPECT P hypoperfusion	*n* = 1 diffuse slow waves	*n* = 1 Aβ_42_↓	*n* = 2 AP, NFTs, α-Synuclein	Guardia-Laguarta et al., 2010; Pera et al., 2013; Sieczkowski et al., 2015
	I716M	*n* = 1 hippocampal atrophy	–	–	*n* = 1 Aβ_42_↓, T-Tau↑, P-Tau↑	–	Blauwendraat et al., 2016
	I716V	*n* = 1 diffuse atrophy	–	–	–	–	Eckman et al., 1997
	V717F	*n* = 1 diffuse atrophy	–	*n* = 1 T-P-O triphasic, delta waves and sharp waves	*n* = 1 Aβ_42_↓, T-Tau -, P-Tau -	–	Zádori et al., 2017
	V717G	*n* = 1 diffuse atrophy	–	–	–	–	Knight et al., 2009
	V717I	*n* = 3 temporal atrophy *n* = 2 hippocampal atrophy *n* = 9 diffuse atrophy *n* = 4 white mater lesion *n* = 1 cerebral infarction	*n* = 1 SPECT F-P-O hypoperfusion *n* = 2 SPECT diffuse hypoperfusion	*n* = 2 diffuse slow waves *n* = 1 diffuse slow waves and occasional theta wave *n* = 1 normal *n* = 1non-specifically abnormal	–	*n* = 2 AP, NFTs *n* = 2 AP, NFTs, LBs	Mullan et al., 1993b; Brooks et al., 1995; Matsumura et al., 1996; Halliday et al., 1997; Ishimaru et al., 2013; Jiao et al., 2014; Zhang et al., 2017; Lloyd et al., 2020; Zhou et al., 2020
	V717L	*n* = 1 P-T atrophy *n* = 3 diffuse atrophy	*n* = 1 SPECT diffuse hypoperfusion	*n* = 1 normal			Murrell et al., 2000; Godbolt et al., 2006; Abe et al., 2012
	T719P	*n* = 1 temporal atrophy					Ghidoni et al., 2009
	M722K	*n* = 1 diffuse atrophy					Wang et al., 2015
	L723P	*n* = 1 diffuse atrophy					Kwok et al., 2000

CT, computed tomography; MRI, magnetic resonance imaging; PET, positron emission tomography; SPECT, single-photon emission computed tomography; EEG, electroencephalography; CSF, cerebrospinal fluid; ^18^F-AV-45, ^18^F-Florbetapir (AV-45/Amyvid); ^18^F-FDG, ^18^F-fluorodeoxyglucose; ^11^C-PIB, ^11^C-labeled Pittsburgh Compound B; AP amyloid plaques; NTFs, neurofibrillary tangles; LBs, Lewy bodies; T-Tau, total-Tau; P-Tau, phospho-Tau; F, frontal; P, parietal; T, temporal; pre C, precuneus; O, occipital.

Cerebrospinal fluid analysis was available in only 12 patients. Almost all cases matched the typical features of AD, the reduced Aβ_42_ levels and the increased levels of total-Tau (T-Tau) and phospho-Tau (P-Tau). However, one with E693G mutation and one with V717F mutation had normal levels of T-Tau and p-Tau. EEG was presented in 12 cases as well. And the results were as follows: 7 showed diffuse slow waves, two were normal, one was a non-specific abnormality, one had an alpha rhythm with disturbance of frontal activity, and one indicated T-P-O triphasic, delta waves, and sharp waves.

A total of 18 brain autopsies were performed in all studies. All the neuropathological findings fulfilled the Consortium to Establish a Registry for Alzheimer’s disease (CERAD) (Mirra et al., 1991) criteria, characterized by amyloid plaques and neurofibrillary tangles. In addition to amyloid plaques and neurofibrillary tangles, patients with I716F mutation have α-Synuclein in their brains, and Lewy bodies (LBs) were also present in patients with V717I mutation.

### The genotype-phenotype correlations

**FIGURE 2:**
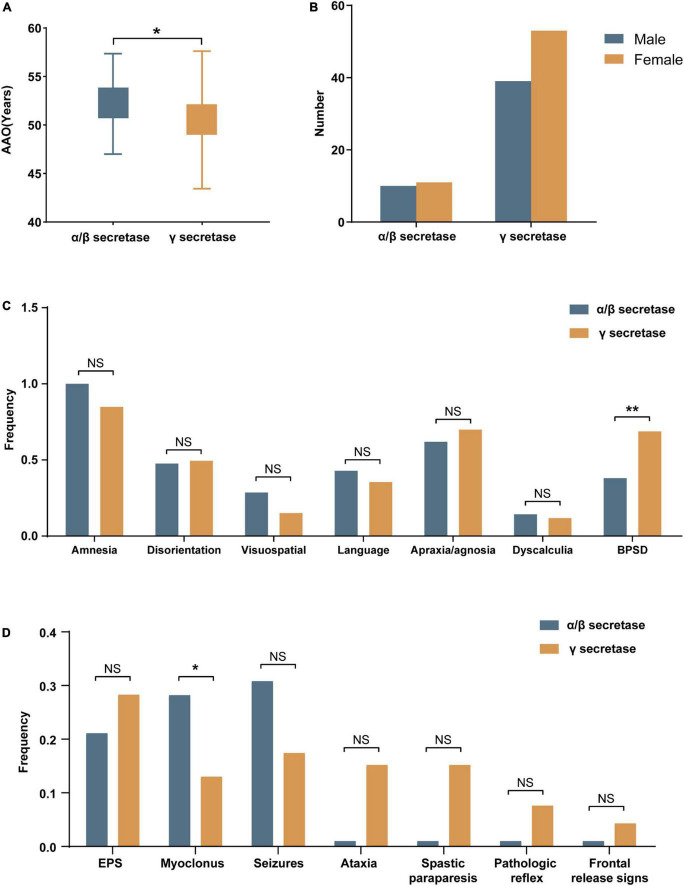
The demographics and the frequencies of clinical features in mutations near α/β secretase and near γ secretase. **(A)** The age at onset (AAO) and **(B)** The gender of mutations near α/β secretase and near γ secretase. **(C)** The frequencies of amnesia and non-amnestic clinical phenotype. **(D)** The frequencies of neurological symptoms (NS, no significance, *p < 0.05, **p < 0.01).

Amyloid protein precursor mutations may alter APP processing, which may in turn diversify phenotypes. We collected the effects on APP processing in pathogenic/likely pathogenic *APP* mutations in Supplementary Table 2. The results showed that the mutation site adjacent to α/β secretase mainly increased the amount of total Aβ, while mutations near γ secretase alter the ratio of Aβ_42_/Aβ_40_. Based on the difference in biochemical results, we divided the pathogenic/likely pathogenic *APP* mutations into two groups: mutations close to α/β secretase and mutations near γ secretase, and compared their clinical features. Atypical mutations such as E693del were ruled out. A total of 130 cases (Complete data not available for all cases) were included in the study of genotype-phenotype correlation analyses, of which 38 were close to α/β secretase, and 92 belonged to the group near γ secretase. The clinical manifestations of the two groups were recorded in Table 2. The phenotype was mostly consistent (Figure 2). However, the AAO in the mutations near the α/β secretase site group was 52.2, a little later than 50.3 in group γ secretase (*P* = 0.049). The frequency of clinical BPSD was also less than that in group γ secretase (*P* = 0.008), and the incidence of myoclonus in the α/β secretase group was higher (*P* = 0.037).

**TABLE 2:** The demographics and the frequencies of clinical features in mutations near α/β secretase and mutations near γ secretase.

	α /β secretase	γ secretase	*P*-value
AAO (years), mean ± SD	52.2 ± 5.2	50.5 ± 7.1	0.049*
Gender (M/F)	10/11	39/53	0.663
Amnesia (*n*, %)	21, 100%	79,85.9%	0.058
**Non-amnestic clinical phenotype (*n*, %)**			
Disorientation	10, 47.6%	46, 50.0%	0.879
Visuospatial disorder	6, 28.6%	14, 15.2%	0.141
Language impairment	9, 42.9%	33, 35.7%	0.527
Apraxia/agnosia	13, 61.9%	65, 70.7%	0.455
Dyscalculia	3, 14.3%	11, 12.0%	0.575
BDSP	8, 38.1%	64, 69.6%	0.008*
**Neurological symptoms (*n*, %)**			
EPS	4, 19.0%	26, 28.3%	0.520
Myoclonus	11, 28.2%	12, 13.0%	0.037*
Seizures	12 30.8%	16, 17.4%	0.088
Ataxia	0, 0.0%	14, 15.2%	0.069
Spastic paraplegia	0, 0.0%	14, 15.2%	0.069
Pathologic reflex	0, 0.0%	7, 8.2%	0.214
Frontal release signs	0, 0.0%	4, 4.3%	0.355

SD, standard deviation; BPSD, behavioral and psychological disorders of dementia; EPS, extrapyramidal symptoms. Clinical features are all shown as numbers and proportions (%). *Statistically significant for *P* < 0.05.

V717I mutation is the most frequently reported and clinically detailed *APP* mutation, and we also compared V717I mutation to all pathogenic/likely pathogenic *APP* mutations. A total of 147 patients were included in genotype-phenotype correlation analyses, with 60 carrying V717I mutation. There was little difference between the two groups except that the visuospatial impairment on V717I was very low, only 3.2% (Supplementary Figure 1). However, when comparing V717I alone with other *APP* mutations (Supplementary Tables 3, 4), we found that patients with the V717I mutation had a later onset and tended to have dyscalculia but had less damage in linguistic and visuospatial regions. V717I mutation carriers also had a higher prevalence of ataxia and spastic paraplegia regarding neurological symptoms (Supplementary Figure 2).

We also performed correlation analysis for clinical phenotypes. Apraxia/agnosia showed a weak positive correlation with language impairment (*r* = 0.308, *p* = 0.001), and BPS was negatively correlated with the visuospatial disorder (*r* = −0.301, *p* = 0.001). And the individuals with ataxia were more likely to have spastic paraplegia (*r* = 0.468, *p* = 1.711 × 10^–7^).

## Discussion

This is the first study to collect clinical data on *APP* mutations defined as pathogenic/likely pathogenic according to ACMG and describe genotype-phenotype correlations of FAD cases with *APP* mutations. Although *APP* mutations are the second most common pathogenic gene for AD, the information on clinical manifestations of *APP* mutations was relatively limited. In general, *APP* mutations are consistent with the typical AD phenotype, even if there are some specific and heterogeneous features. And there are also some differences in clinical manifestations between *APP* mutations and *PSEN1/PSEN2*.

Previous studies verified that the pedigrees with *APP* mutations have an earlier mean AAO than those with *PSEN2* mutations but later than families with *PSEN1* mutations (Mullan et al., 1993a; Jayadev et al., 2010). A study of clinical phenotypic and genetic association analysis of autosomal dominant FAD in the UK demonstrated that the mean AAO of *PSEN1* and *APP* mutations was 43.6 and 50.4, respectively (Ryan et al., 2016). The AAO of our study was 50.7, the same as the AAO of *APP* mutations in their research and later than that of *PSEN1* mutations. *APOE* ε4 is a well-established risk factor for LOAD. *APOE* ε4 carriers had a significantly earlier AAO of AD than ε4 non-carriers. A study showed a decrease in 3.02 years in AAO for each unit increase in the number of ε4 alleles (Sorbi et al., 1995; Thambisetty et al., 2013). However, the *APOE* allele only affected the AAO of V717I mutation in our research. The limited data and confounding factors interfered with our ability to analyze the correlations between AAO and *APOE* alleles in other pathogenic/likely pathogenic *APP* mutations other than V717I. Moreover, since AAO measurement is usually retrospective and prone to recall bias, the accuracy of AAO itself remains to be determined.

Our results indicated that *APP* pathogenic/likely pathogenic mutations all have the following clinical characteristics. First, most cases start with amnesia. Second, the disease progresses relatively slowly. Third, patients rarely exhibit pure progressive amnesia and usually present with impairment in multiple cognitive domains. Fourth, BPSD frequently occurs in the progression of dementia and manifests in various forms, among which affective symptoms represented by depression and anxiety are the most common. Fifth, the neuroimaging, CSF biochemical, and neuropathological findings are typical in most cases. The clinical phenotype of *APP* mutations is similar to the SAD (Swearer et al., 1992), but some specificity and heterogeneity remain. For instance, patients with *APP* mutations are more likely to have apraxia/agnosia and perform worse than LOAD (Koedam et al., 2010; Smits et al., 2012).

Regarding BPSD, anxiety and depression are the most common in *APP* mutations, while depression and irritability are more frequent in LOAD (Gumus et al., 2021). The higher incidence of anxiety and depression with less irritability is also well represented in *PSEN*1 and *PSEN2* (Kaiser et al., 2014; Panegyres and Chen, 2014), but they tend to have more hallucinations and delusions compared with *APP* mutations (Larner and Doran, 2006; Canevelli et al., 2014). The consistency and heterogeneity of the three pathogenic genes in BPSD may be related to both AAO and genes. In order to clarify whether there is a connection, it is necessary to pay more attention to the relationship between each subtype of BPSD in patients with dementia and AAO and genetics in future research. In addition, the disease progression of early-onset FAD is faster than that of late-onset SAD, with more extensive cognitive impairment and higher mortality (Jacobs et al., 1994), and this was broadly to our study. While in our study, the disease duration was not as short as previously reported, the average time from onset to death is only 6.6 years (Vermunt et al., 2019; Brück et al., 2021).

Similar to the non-amnestic phenotypes, neurological symptoms have higher morbidity in early-onset FAD than in late-onset SAD (Bateman et al., 2011), especially in pedigrees with AAO <40 years (Ryan and Rossor, 2010). Cases with *APP*, *PSEN1*, or *PSEN2* mutations in FAD also differ in neurological symptoms. A study in autosomal dominant familial AD reported that apart from seizures and myoclonus, patients with *PSEN1* mutations can present with other neurological symptoms while patients with *APP* mutations do not (Ryan et al., 2016). Differently, patients with *APP* mutation in this study also presented with EPS, spastic paraparesis, pathologic reflex, and ataxia. EPS is even more frequently in *APP* than in *PSEN1*, and the proportions of patients with myoclonus or seizures are the same. In the same study, Ryan et al. (2016) also pointed out that individuals with myoclonus tended to develop more seizures than those without myoclonus, yet this was not confirmed in our research. Instead, we found that patients with EPS or ataxia were more likely to have spastic paraplegia. Moreover, we also found that the severity of neurological symptoms would gradually increase as dementia progresses (Vöglein et al., 2019). Similarly, patients with neurological symptoms such as EPS experience a faster cognitive decline (Chui et al., 1994).

We conducted genotype-phenotype correlation analysis of *APP* mutations and found that biochemical differences due to mutations can lead to differences in clinical manifestations, which can occur in AAO, non-demented symptoms, and neurological symptoms. However, the clinical data of the patients we obtained were somewhat limited, which may have affected our interpretation of the results. Future studies need to focus more on the heterogeneity of clinical manifestations caused by differences in APP processing, Aβ amount, and the ratio of Aβ_42_/Aβ_40_, which may offer a better understanding of amyloid pathways in AD. Furthermore, the differences between V717I and all pathogenic/likely pathogenic *APP* mutations suggest that each *APP* mutation may have the diverse characteristic. For example, V715M and V717L have an earlier AAO than V717I. Some *APP* mutations had visuospatial and language impairments in addition to amnesia, while others had minor damage in these cognitive areas but showed more dyscalculia (e.g., V717I). And mutations in exon 16 are rare to show neurological symptoms compared to mutations in exon 17. Therefore, attention to the clinical manifestations of each mutation is highly warranted.

For the most reported V717I mutation, there was consistency in the pattern of symptoms between cases. There is a cognitive decline initially, with visuospatial impairment, disorientation, and language impairment. Dyscalculia, agnosia/apraxia and neurological symptoms occur as the disease progresses. However, there are differences in clinical manifestations among pedigrees (Mullan et al., 1993b). For instance, the APPV717I mutation in the Chinese population mainly manifests as affective symptoms, executive dysfunction and disorientation in the early stage, and spastic paraparesis and ataxia in the late stage are more common (Zhang et al., 2017). In addition, we also found differences in AAO and clinical manifestations within the same family. This may be due to variability in the expression of these mutations or related to other genetic or epigenetic factors (Román et al., 2019).

Although most pathogenic/likely pathogenic *APP* mutations are complete penetrance, some have been demonstrated to behave as incomplete dominant. A Caucasian woman with the homozygous mutation D678N had memory difficulties early in her third decade and developed full-blown clinical symptoms 10 years later. Her heterozygous affected siblings were generally diagnosed with dementia in their 60 s (Mastromoro et al., 2019). We also found asymptomatic carriers reported in some other pathogenic/likely pathogenic *APP* mutations, which may be due to incomplete dominant mutations or maybe just because of the individual differences as well as environmental factors that lead to a later onset in those carriers, and the researchers did not follow them up. And unlike other *APP* mutations (Zhou et al., 2011; Huang et al., 2019), A673V is a particular mutation caused by recessive homozygous mutation. It has a high amyloidosis effect in the homozygous state and an anti-amyloidosis effect in the heterozygous state (Di Fede et al., 2009). In addition, different mutations in the same site of *APP* can lead to different diseases. For example, E693K (Bugiani et al., 2010), E693Q (Wattendorff et al., 1982; Luyendijk and Bots, 1986) can lead to Cerebral Amyloid Angiopathy (CAA), E693G, and E693del lead to AD, D694N (Grabowski et al., 2001) mutation near 693 can lead to vascular dementia (VD). All of these suggest that there may be some discrepancy in the structure and toxicity of the mutant product Aβ, or other mechanisms related to other APP cleavage products besides Aβ such as APP intracellular domain (AICD). It is essential to use these atypical mutations as tools to study the pathogenesis of AD.

The limited clinical evidence makes it difficult to conduct further genotype-phenotype association analyses, especially to evaluate the clinical manifestations of different mutations at the same locus. And the reliability of the clinical data we collected remains to be determined. We are not able to be sure whether the specific manifestations unreported were because they were really absent or emerged after the study, or were just not mentioned in the articles. Besides, we only analyzed *APP* mutations and did not compare them with the other two pathogenic genes.

In conclusion, we collected the clinical data from patients with pathogenic/likely pathogenic *APP* mutations and performed an analysis of genotype-phenotypic association, which may help better understand the relationship between genotype and phenotype and may be beneficial for clinical practice prediction, diagnosis, and genetic counseling.

## Data availability statement

Publicly available datasets were analyzed in this study. This data can be found here: https://www.alzforum.org/mutations.

## Author contributions

YL and XX contributed to the conception and design of the study. YL collected the data, performed the statistical analysis, and wrote the first draft of the manuscript. All authors contributed to manuscript revision, read, and approved the submitted version.

## Footnotes

1
https://www.alzforum.org/mutations
