# Supplementary material for: Clinical characteristics and genotype-phenotype correlation analysis of familial Alzheimer’s disease patients with pathogenic/likely pathogenic amyloid protein precursor mutations
Source: Front Aging Neurosci. 2022 Oct 14;14:1013295. doi: 10.3389/fnagi.2022.1013295 (PMC9616047; doi:10.3389/fnagi.2022.1013295)
Supplement: Supplementary file 1 [file Data_Sheet_1.docx]

**Supplementary Table 1.** The demographic and clinical characteristics in pathogenic/likely pathogenic *APP* mutations.

| **Exon** | **Mutation** | **Families and Origin** | **Affected** | **Gender, M/F** | **Mean AAO, y (range)** | **Onset symptom** | **Amnesia** | **Non-amnestic clinical phenotype** | **neurological symptoms** | | **BPSD** | ***APOE*** | **Non-dementia symptoms** | | **Disease duration**  **(range)** | **References** |
| --- | --- | --- | --- | --- | --- | --- | --- | --- | --- | --- | --- | --- | --- | --- | --- | --- |
| 16 | KM670/671NL | n=3 Sweden | 43 | - | 53.8  (44-61) | n=28 amnesia  n=4 BPSD | 28 | n=9 Disorientation  n=14 Language impairment  n=5 BPSD | n=10 myoclonus  n=10 seizures | | Delusion/  hallucination  depression  aggressiveness | n=2 ε2/ε3  n=1 ε4/ε4 | - | | 7.9  (3-13) | [1-3] |
|  | A673V | n=1 Italy | 1 | 1/0 | 36.0 | -- | 1 | Language impairment  Apraxia/ agnosia  BPSD | myoclonus  Spastic paraplegia | | depression  aggressiveness | n=1 ε3/ε3 | - | | - | [4] |
|  | D678H | n=3 Taiwan | 6 | 2/4 | 51.0  (47-55) | n=5 amnesia | 6 | n=5 Disorientation  n=2 Visuospatial disorder  n=1 Language impairment  n=2 Apraxia/ agnosia  n=5 BPSD | - | | Delusions/  hallucination  anxiety | n=5 ε3/ε3 | - | | - | [5, 6] |
|  | D678N | n=1 Japan  n=2 China | 4 | 1/3 | 56.0  (42-64) | n=1 amnesia | 4 | n=1 Disorientation  n=1 Visuospatial disorder  n=1 Language impairment  n=3 Apraxia/ agnosia  n=2 BPSD | n=2 EPS | | apathy  irritability  aggressiveness | n=1 ε2/ε3 | - | | - | [7-9] |
|  | E682K | n=1 Belgium | 1 | 0/1 | 47 | n=1 depression | 1 | n=1 Language impairment  n=1 Apraxia/ agnosia | - | | depression | n=1 ε3/ε3 | - | | - | [10] |
|  | K687N | n=1 German  n=1 China | 2 | 0/2 | 51.5  (51-52) | - | 2 | n=1 Disorientation  n=2 Visuospatial disorder  n=2 Language impairment  n=1 Apraxia/ agnosia  n=1 Dyscalculia | - | | - | n=1 ε2/ε3 | - | | - | [11, 12] |
|  | K687Q | n=2 China | 2 | 2/0 | 57.5  (52-63) | n=2 amnesia | 2 | n=1 Visuospatial disorder  n=1 Dyscalculia | - | | - | n=1 ε2/ε3  n=1 ε3/ε3 | - | | - | [13] |
| 17 | A692G | n=1 Dutch  n=1 UK | 5 | 4/1 | 47.4  (39-54) | - | 5 | n=3 Disorientation  n=1 Visuospatial disorder  n=3 Language impairment  n=4 Apraxia/ agnosia  n=1 Dyscalculia  n=1 BPSD | n=2 EPS  n=1 myoclonus  n=2 seizures | | depression | - | n=1 headache | | 9.4  (8-11) | [14, 15] |
|  | E693del | n=3 Japan | 6 | 1/5 | 51.3  (35-59) | n=3 amnesia | 5 | n=1 Disorientation  n=1 Visuospatial disorder  n=1 Apraxia/ agnosia  n=4 BPSD | n=2 EPS  n=3 ataxia  n=3 Spastic paraplegia  n=2 pathologic reflex | | mutism  depression | n=3 ε3/ε3 |  | | - | [16-19] |
|  | E693G | n= 1 Sweden  n=1 USA | 14 | 4/2 | 57.4  (52-65) | n=9 amnesia  n=1 headache | 10 | n=10 Disorientation  n=5 Language impairment  n=6 Apraxia/ agnosia  n=9 BPSD | n=5 EPS  n=3 myoclonus  n=1 seizures | | Delusions/  hallucination  anxiety  suspiciousness  mutism  apathy  aggressiveness  depression | - | n=1 headache  fatigue  n=2 sleep disturbance | | 8.6  (3-16) | [20, 21] |
|  | T714A | n=1 Denmark  n=1 Iran  n=1 Poland | 5 | 1/4 | 49.2  (44-58) | n=2 amnesia  n=1 distraction | 5 | n=2 Disorientation  n=2 Visuospatial disorder  n=5 Language impairment  n=3 Apraxia/ agnosia  n=4 BPSD | n=1 EPS  n=1 seizures | | delusions  anxiety  aggression  depression | - |  | | - | [22-25] |
|  | T714I | n=1 Austria  n=1 USA | 3 | 2/1 | 34 | n=1 amnesia  n=1 visual dysfunction | 3 | n=2 Disorientation  n=2 Visuospatial disorder  n=1 Apraxia/ agnosia  n=3 BPSD | n=2 EPS  n=1 myoclonus  n=2 seizures  n=1 spastic paraplegia  n=1 pathologic reflex  n=1 frontal release signs | | aggression  depression | n=1 ε3/ε3 |  | | 4.0  (3-5) | [26, 27] |
|  | V715A | n=1 German  n=1 Poland | 2 | 0/2 | 45.0  (42-48) | n=1 amnesia | 2 | n=2 Visuospatial disorder  n=2 Language impairment  n=1 Apraxia/ agnosia | - | | - | - |  | | - | [25, 28] |
|  | V715M | n=1 France  n=1 Korea  n=1 China | 3 | 2/1 | 44.0  (41-47) | n=1 amnesia | 3 | n=1 Disorientation  n=1 Visuospatial disorder  n=2 Language impairment  n=3 Apraxia/ agnosia  n=1 BPSD | n=1 EPS  n=1 seizures | | apathy | n=1 ε3/ε3 | n=1 blepharospasm | | 10.0  (6-14) | [29-31] |
|  | I716F | n=1 Austria  n=1 Spain | 2 | 2/0 | 38.0  (31-45) | n=1 amnesia  n=1 depression | 2 | n=1 Disorientation  n=1 Visuospatial disorder  n=2 Language impairment  n=1 Apraxia/ agnosia  n=1 BPSD | n=1 EPS  n=1 myoclonus  n=2 seizures  n=1 ataxia | | depression | n=1 ε3/ε3 |  | | 7.0  (3-10) | [32-34] |
|  | I716M | n=1 German | 1 | 1/0 | 64 | - | 1 | n=1 Disorientation  n=1 Apraxia/ agnosia  n=1 BPSD | - | | avolition | - |  | | - | [35] |
|  | I716T | n=1 | 1 | 1/0 | - | - | 1 | n=1 BPSD | - | | - | - |  | | - | [36] |
|  | I716V | n=1 USA | 1 | 0/1 | 53 | - | 1 | n=1 Visuospatial disorder  n=1 Language impairment | - | | - | - |  | | - | [37] |
|  | V717F | n=1 Hungary | 2 | 0/1 | - | n=1 amnesia | 2 | n=1 Disorientation  n=2 Language impairment  n=1 Apraxia/ agnosia  n=1 BPSD | n=1 EPS  n=1 myoclonus  n=2 seizures | | aggression | - |  | | - | [38] |
|  | V717G | n=1 UK | 1 | 1/0 | 44 | n=1 amnesia | 1 | - | - | | - | - |  | | - | [39] |
|  | V717I | n=2 Australia  n=9 China  n=3 Japan  n=1 UK  n=2 USA | 69 | 27/41 | 52.6  (37-62) | n=15 amnesia  n=1 disorientation  n=1 dyscalculia  n=1 headache  n=2 vertigo | 48 | n=36 Disorientation  n=2 Visuospatial disorder  n=15 Language impairment  n=45 Apraxia/ agnosia  n=11 Dyscalculia  n=44 BPSD | n=19 EPS  n=8 myoclonus  n=7 seizures  n=13 ataxia  n=13 spastic paraplegia  n=3 pathologic reflex  n=3 frontal release signs | Delusions/  hallucination  depression  aggression  anxiety  Irritability  apathy | | n=1 ε2/ε3  n=2 ε2/ε4  n=20 ε3/ε3  n=9 ε3/ε4  n=4 ε4/ε4 | | n=1  sleep disturbance  n=2 vertigo  n=2 headache  n=3 cerebrovascular accident  n=1 hydrocephalus | 9.9  (3-18) | [40-48] |
|  | V717L | n=1 UK  n=1 USA  n=1 Japan | 6 | 2/3 | 42.8  (35-51) | n=3 amnesia  n=1 distraction  n=1  working executive dysfunction | 6 | n=1 Disorientation  n=3 Visuospatial disorder  n=2 Language impairment  n=5 Apraxia/ agnosia  n=4 BPSD | n=1 EPS  n=1 myoclonus  n=1 seizures  n=3 pathologic reflex | | hallucination  aggression  anxiety  Irritability  depression | n=2 ε3/ε3 |  | | - | [49-51] |
|  | T719N | - | - | - | - | - | - | - | - | | - | - |  | | - | - |
|  | T719P | n=1 Italy | 1 | 0/1 | 43 | n=1 amnesia | 1 | n=1 Apraxia/ agnosia  n=1 BPSD | n=1 EPS | | Irritability  aggression | n=1 ε3/ε3 |  | | - | [52] |
|  | M722K | n=1 China | 1 | 0/1 | 42 | n=1 amnesia | 1 | n=1 Disorientation  n=1 Language impairment  n=1 Apraxia/ agnosia  n=1 BPSD | - | | depression | n=1 ε3/ε4 |  | | - | [53] |
|  | L723P | n=1 Australia | 1 | 1/0 | 45 | - | 1 | n=1 Language impairment  n=1 Apraxia/ agnosia  n=1 BPSD | - | | hallucination  persecutory delusions  aggression | - |  | | - | [54] |
|  | K724N | n=1 Belgium | 1 | 0/1 | 55 | n=1 amnesia | 1 | n=1 Apraxia/ agnosia  n=1 BPSD | - | | anxiety  depression  apathy | n=1 ε3/ε4 |  | | - | [55] |

Abbreviations: AAO, age at onset; BPSD, behavioral and psychological disorders of dementia; EPS, extrapyramidal symptoms

**Supplementary Table 2.** The effects on APP processing in pathogenic/likely pathogenic *APP* mutations.

| **Exon** | **Mutation** | **APP processing** | | | | | **References** | |  |
| --- | --- | --- | --- | --- | --- | --- | --- | --- | --- |
|  |  | **Aβ_40_** | **Aβ_42_** | **Aβ_42_/Aβ_40_** | **Total Aβ** | **Source** | |  | |
| 16 | KM670/671NL | Increased 7x  Increased 3.4x | Increased 8x  Increased 3x | Not increased  Not increased | Increased 4x~5x  Increased 3x | Hek293  N2A | | [30, 53, 56] | |
|  | A673V | Slightly increased  Increased 1.7x | Slightly increased  Increased 2x | Not increased  Not increased | Slightly increased  Increased 1.8x | COS7  CHO | | [4, 57] | |
|  | D678H | Slightly increased | Increased 2.4x | Increased 1.7x | Increased | Hek293 | | [5, 57] | |
|  | D678N |  | Not increased |  | Not increased | Hek293 | | [57] | |
|  | E682K | Slightly increased  Increased 1.7x  Increased 1.7x | Increased 3x  Increased 2.5x  Increased 3x | Increased  Slightly increased  Increased 1.7x | Increased  Increased 1.8x  Increased 2x | Hek293  CHO  primary neurons | | [10, 58] | |
|  | K687N | Increased 2x | Increased 2x | Not increased | Increased 2x | Hek293 | | [11] | |
|  | K687Q |  |  |  |  |  | |  | |
| 17 | A692G | Increased 1.6x  Increased 1.8x  Increased 2x | Increased 2x  Increased 2x  Increased 2x | Slightly increased  Not increased  Not increased | Increased 1.7x  Increased 1.8x  Increased 2x | Hek293  CHO  primary neurons | | [10, 56] | |
|  | E693del | Decreased 40% | Decreased 40% | Not increased | Decreased | Hek293 | | [16] | |
|  | E693G | Not increased | Slightly decreased | Decreased 20% | Not increased | Hek293 | | [56] | |
|  | T714A |  |  |  |  |  | |  | |
|  | T714I | Decreased 68%  Decreased 80% | Increased 3.5x  Increased 1.6x | Increased 11x  Increased 8.2x |  | Hek293T  primary neurons | | [26, 59] | |
|  | V715A | Decreased 45% | Increased | Increased 3x  Increased 4x |  | primary neurons  Hek293 | | [28, 59] | |
|  | V715M | Decreased 50%  Decreased 70% | Not increased | Increased 1.7x  Increased 4x | Decreased 50% | Hek293  primary neurons | | [30, 59] | |
|  | I716F | Decreased 50% | Increased 2x | Increased 4x | Decreased 50% | CHO | | [32] | |
|  | I716M |  |  |  |  |  | |  | |
|  | I716T | Decreased 20% | Increased 2.7x | Increased 12x | Not increased | CHO | | [60] | |
|  | I716V | Not increased  Not increased  Increased 2.4x | Increased  Increased 1.7x  Increased 3x | Increased 1.9x  Increased 1.6x  Slightly increased | Not increased  Increased 2.4x | primary neurons  Hek293  CHO | | [37, 59] | |
|  | V717F | Decreased 40% | Increased 1.8x | Increased 3x | Decreased 30% | Hek293 | | [56] | |
|  | V717G | Decreased | Increased | Increased |  | COS | | [61] | |
|  | V717I | Decreased 25%  Decreased 50% | Increased 2.7x  Slightly increased | Increased 1.8x  Increased 1.9x  Increased 3x | Not increased | Hek293T  primary neurons  CHO | | [26, 32, 37, 56, 59] | |
|  | V717L | Decreased 25% | Increased | Increased 4x |  | primary neurons | | [59] | |
|  | T719N | Slightly decreased | Increased 5x | Increased 5x |  | N2A | | [62] | |
|  | T719P |  |  |  |  |  | |  | |
|  | M722K | Slightly decreased | Slightly increased | Increased 1.7x |  | N2A | | [53] | |
|  | L723P |  |  | Increased 1.9x |  | CHO | | [54] | |
|  | K724N | Decreased 30% | Increased 2x | Increased 2.6x  Increased 3x |  | Hek293T  primary neurons | | [55] | |

**Supplementary Table 3.** The demographics and the frequencies of clinical features in V717I mutation and other mutations.

|  | V717I | All *APP* mutations | P Value |
| --- | --- | --- | --- |
| AAO (years), mean ± SD | 52.9 ± 5.5 | 51.1 ± 6.8 | 0.106 |
| Gender (M/F) | 26/36 | 54/71 | 0.869 |
| Amnesia (n, %) | 49, 79.0% | 115, 88.5% | 0.083 |
| Non-amnestic clinical phenotype (n, %) |  |  |  |
| Disorientation | 36, 58.3% | 66, 35.8% | 0.344 |
| Visuospatial disorder | 2, 3.2% | 21, 16.2% | 0.010 * |
| Language impairment | 15, 24.2% | 47, 36.2% | 0.097 |
| Apraxia/ agnosia | 45, 72.6% | 85, 65.4% | 0.319 |
| Dyscalculia | 11, 17.7% | 14, 10.8% | 0.179 |
| BDSP | 44, 71.0% | 85, 65.4% | 0.441 |
| Neurological symptoms (n, %) |  |  |  |
| EPS | 18, 22.2% | 37, 29.1% | 0.958 |
| Myoclonus | 9, 14.8% | 26, 17.7% | 0.607 |
| Seizures | 8, 13.1% | 29, 19.7% | 0.256 |
| Ataxia | 13, 21.3% | 17, 13.4% | 0.165 |
| Spastic paraplegia | 13, 21.3% | 17, 13.4% | 0.165 |
| Pathologic reflex | 3, 4.9% | 9, 7.1% | 0.569 |
| Frontal release signs | 3, 4.9% | 4, 3.1% | 0.549 |

SD, standard deviation; BPSD, behavioral and psychological disorders of dementia; EPS, extrapyramidal symptoms. Clinical features are all shown as numbers and proportions (%). *, statistically significant for P < 0.05

**Supplementary Table 4.** The demographics and the frequencies of clinical features in V717I mutation and other mutations.

|  | V717I | Other variants | P Value |
| --- | --- | --- | --- |
| AAO (years), mean ± SD | 52.9 ± 5.5 | 51.2 ± 6.9 | 0.087 |
| Gender (M/F) | 26/36 | 28/35 | 0.777 |
| Amnesia (n, %) | 49, 79.0% | 66,97.1% | 0.001 * |
| Non-amnestic clinical phenotype (n, %) |  |  |  |
| Disorientation | 36, 58.3% | 30, 44.1% | 0.112 |
| Visuospatial disorder | 2, 3.2% | 19, 27.9% | 1.310×10^-4^ * |
| Language impairment | 15, 24.2% | 32, 47.1% | 0.007 * |
| Apraxia/ agnosia | 45, 72.6% | 40, 58.8% | 0.100 |
| Dyscalculia | 11, 17.7% | 3, 4.4% | 0.014* |
| BDSP | 44, 71.0% | 41, 60.3% | 0.201 |
| Neurological symptoms (n, %) |  |  |  |
| EPS | 18, 22.2% | 19, 28.8% | 0.929 |
| Myoclonus | 9, 14.8% | 17, 19.8% | 0.433 |
| Seizures | 8, 13.1% | 21, 24.4% | 0.090 |
| Ataxia | 13, 21.3% | 4, 4.7% | 0.012 * |
| Spastic paraplegia | 13, 21.3% | 4, 4.7% | 0.012 * |
| Pathologic reflex | 3, 4.9% | 6, 7.0% | 0.360 |
| Frontal release signs | 3, 4.9% | 1, 1.2% | 0.273 |

SD, standard deviation; BPSD, behavioral and psychological disorders of dementia; EPS, extrapyramidal symptoms. Clinical features are all shown as numbers and proportions (%). *, statistically significant for P < 0.05


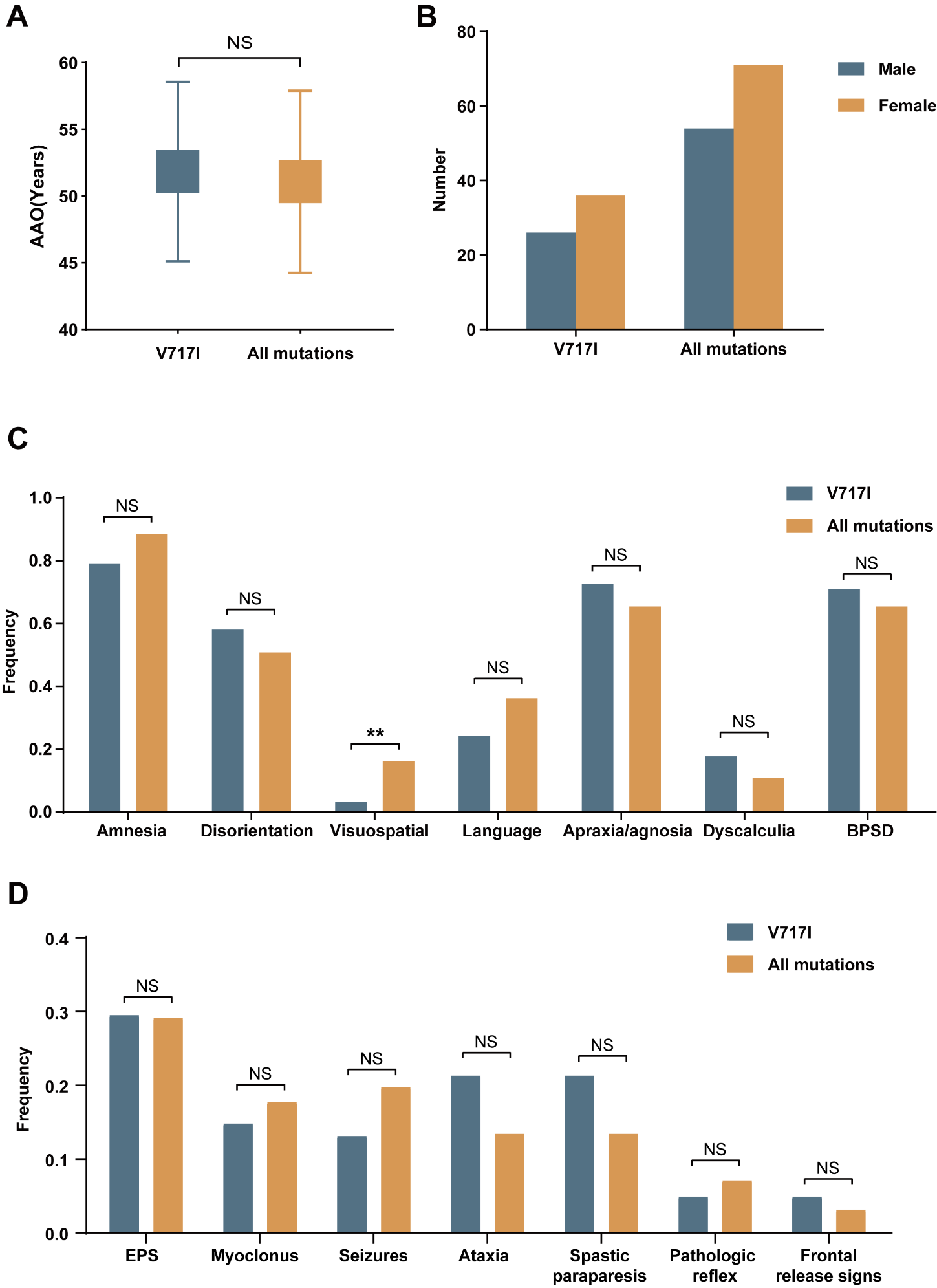


**Supplementary figure 1. The demographics and the frequencies of clinical features in V717I mutation and all *APP* mutations.** A. The AAO and B. The gender of V717I mutation and all *APP* mutations. C. The frequencies of amnesia and non-amnestic clinical phenotype. D. The frequencies of neurological symptoms. (* p < 0.05, ** p < 0.01, *** p < 0.001).


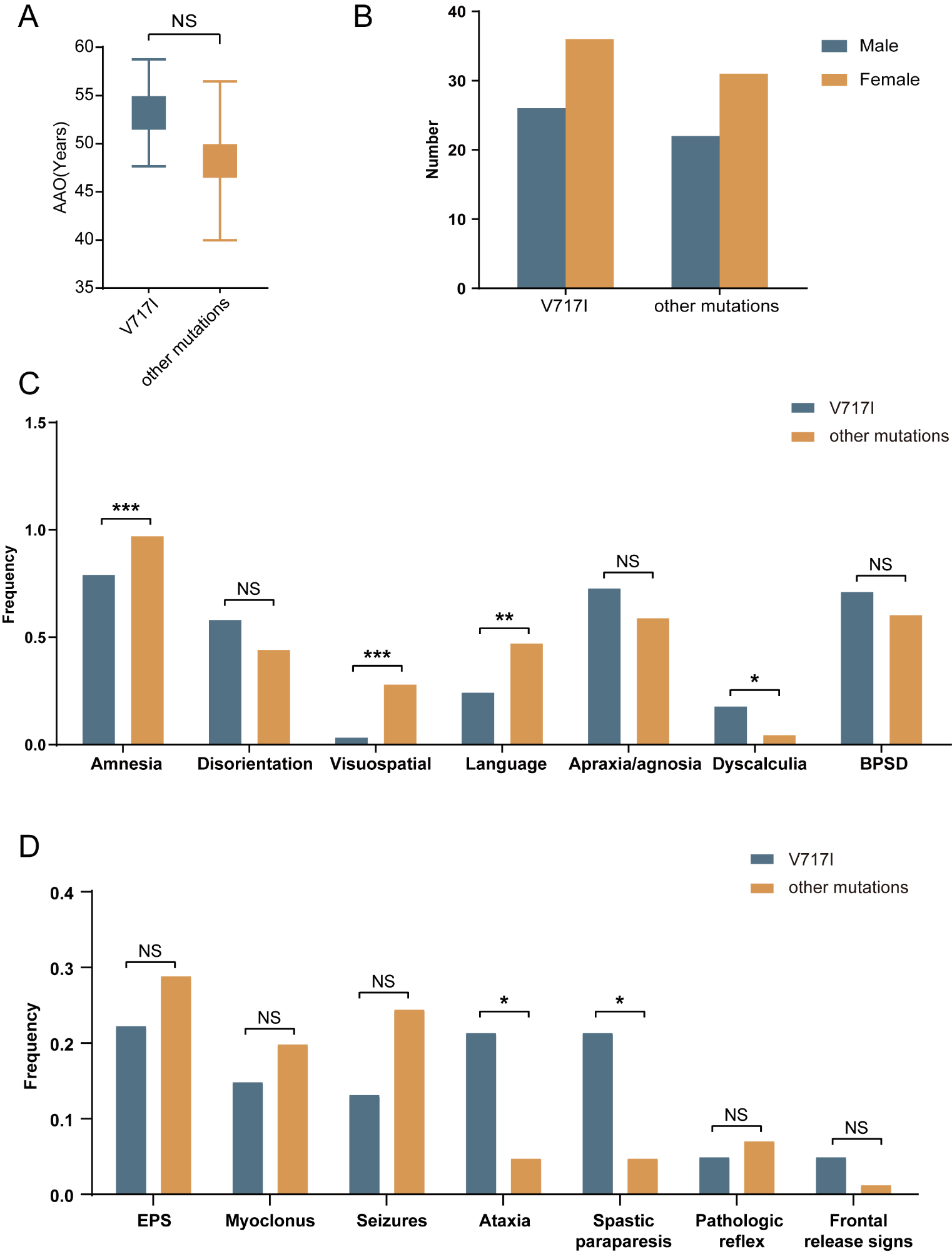


**Supplementary figure 2. The demographics and the frequencies of clinical features in V717I mutation and other mutations.** A. The AAO and B. The gender of V717I mutation and other mutations. C. The frequencies of amnesia and non-amnestic clinical phenotype. D. The frequencies of neurological symptoms. (* p < 0.05, ** p < 0.01, *** p < 0.001).

**Supplementary References**

1. Mullan, M.; Crawford, F.; Axelman, K.; Houlden, H.; Lilius, L.; Winblad, B.; Lannfelt, L., A pathogenic mutation for probable Alzheimer's disease in the APP gene at the N-terminus of beta-amyloid. *Nat Genet* **1992,** 1, (5), 345-7.

2. Bogdanovic, N.; Corder, E.; Lannfelt, L.; Winblad, B., APOE polymorphism and clinical duration determine regional neuropathology in Swedish APP(670, 671) mutation carriers: implications for late-onset Alzheimer's disease. *J Cell Mol Med* **2002,** 6, (2), 199-214.

3. Axelman, K.; Basun, H.; Winblad, B.; Lannfelt, L., A large Swedish family with Alzheimer's disease with a codon 670/671 amyloid precursor protein mutation. A clinical and genealogical investigation. *Arch Neurol* **1994,** 51, (12), 1193-7.

4. Di Fede, G.; Catania, M.; Morbin, M.; Rossi, G.; Suardi, S.; Mazzoleni, G.; Merlin, M.; Giovagnoli, A. R.; Prioni, S.; Erbetta, A.; Falcone, C.; Gobbi, M.; Colombo, L.; Bastone, A.; Beeg, M.; Manzoni, C.; Francescucci, B.; Spagnoli, A.; Cantù, L.; Del Favero, E.; Levy, E.; Salmona, M.; Tagliavini, F., A recessive mutation in the APP gene with dominant-negative effect on amyloidogenesis. *Science* **2009,** 323, (5920), 1473-7.

5. Chen, W. T.; Hong, C. J.; Lin, Y. T.; Chang, W. H.; Huang, H. T.; Liao, J. Y.; Chang, Y. J.; Hsieh, Y. F.; Cheng, C. Y.; Liu, H. C.; Chen, Y. R.; Cheng, I. H., Amyloid-beta (Aβ) D7H mutation increases oligomeric Aβ42 and alters properties of Aβ-zinc/copper assemblies. *PLoS One* **2012,** 7, (4), e35807.

6. Huang, C. Y.; Hsiao, I. T.; Lin, K. J.; Huang, K. L.; Fung, H. C.; Liu, C. H.; Chang, T. Y.; Weng, Y. C.; Hsu, W. C.; Yen, T. C.; Huang, C. C., Amyloid PET pattern with dementia and amyloid angiopathy in Taiwan familial AD with D678H APP mutation. *J Neurol Sci* **2019,** 398, 107-116.

7. Mao, C.; Li, J.; Dong, L.; Huang, X.; Lei, D.; Wang, J.; Chu, S.; Liu, C.; Peng, B.; Román, G. C.; Cui, L.; Gao, J., Clinical Phenotype and Mutation Spectrum of Alzheimer's Disease with Causative Genetic Mutation in a Chinese Cohort. *Curr Alzheimer Res* **2021,** 18, (3), 265-272.

8. Han, L. H.; Xue, Y. Y.; Zheng, Y. C.; Li, X. Y.; Lin, R. R.; Wu, Z. Y.; Tao, Q. Q., Genetic Analysis of Chinese Patients with Early-Onset Dementia Using Next-Generation Sequencing. *Clin Interv Aging* **2020,** 15, 1831-1839.

9. Wakutani, Y.; Watanabe, K.; Adachi, Y.; Wada-Isoe, K.; Urakami, K.; Ninomiya, H.; Saido, T. C.; Hashimoto, T.; Iwatsubo, T.; Nakashima, K., Novel amyloid precursor protein gene missense mutation (D678N) in probable familial Alzheimer's disease. *J Neurol Neurosurg Psychiatry* **2004,** 75, (7), 1039-42.

10. Zhou, L.; Brouwers, N.; Benilova, I.; Vandersteen, A.; Mercken, M.; Van Laere, K.; Van Damme, P.; Demedts, D.; Van Leuven, F.; Sleegers, K.; Broersen, K.; Van Broeckhoven, C.; Vandenberghe, R.; De Strooper, B., Amyloid precursor protein mutation E682K at the alternative β-secretase cleavage β'-site increases Aβ generation. *EMBO Mol Med* **2011,** 3, (5), 291-302.

11. Kaden, D.; Harmeier, A.; Weise, C.; Munter, L. M.; Althoff, V.; Rost, B. R.; Hildebrand, P. W.; Schmitz, D.; Schaefer, M.; Lurz, R.; Skodda, S.; Yamamoto, R.; Arlt, S.; Finckh, U.; Multhaup, G., Novel APP/Aβ mutation K16N produces highly toxic heteromeric Aβ oligomers. *EMBO Mol Med* **2012,** 4, (7), 647-59.

12. Xu, Y.; Liu, X.; Shen, J.; Tian, W.; Fang, R.; Li, B.; Ma, J.; Cao, L.; Chen, S.; Li, G.; Tang, H., The Whole Exome Sequencing Clarifies the Genotype- Phenotype Correlations in Patients with Early-Onset Dementia. *Aging Dis* **2018,** 9, (4), 696-705.

13. Jiang, B.; Zhou, J.; Li, H. L.; Chen, Y. G.; Cheng, H. R.; Ye, L. Q.; Liu, D. S.; Chen, D. F.; Tao, Q. Q.; Wu, Z. Y., Mutation screening in Chinese patients with familial Alzheimer's disease by whole-exome sequencing. *Neurobiol Aging* **2019,** 76, 215.e15-215.e21.

14. Brooks, W. S.; Kwok, J. B.; Halliday, G. M.; Godbolt, A. K.; Rossor, M. N.; Creasey, H.; Jones, A. O.; Schofield, P. R., Hemorrhage is uncommon in new Alzheimer family with Flemish amyloid precursor protein mutation. *Neurology* **2004,** 63, (9), 1613-7.

15. Cras, P.; van Harskamp, F.; Hendriks, L.; Ceuterick, C.; van Duijn, C. M.; Stefanko, S. Z.; Hofman, A.; Kros, J. M.; Van Broeckhoven, C.; Martin, J. J., Presenile Alzheimer dementia characterized by amyloid angiopathy and large amyloid core type senile plaques in the APP 692Ala-->Gly mutation. *Acta Neuropathol* **1998,** 96, (3), 253-60.

16. Tomiyama, T.; Nagata, T.; Shimada, H.; Teraoka, R.; Fukushima, A.; Kanemitsu, H.; Takuma, H.; Kuwano, R.; Imagawa, M.; Ataka, S.; Wada, Y.; Yoshioka, E.; Nishizaki, T.; Watanabe, Y.; Mori, H., A new amyloid beta variant favoring oligomerization in Alzheimer's-type dementia. *Ann Neurol* **2008,** 63, (3), 377-87.

17. Kutoku, Y.; Ohsawa, Y.; Kuwano, R.; Ikeuchi, T.; Inoue, H.; Ataka, S.; Shimada, H.; Mori, H.; Sunada, Y., A second pedigree with amyloid-less familial Alzheimer's disease harboring an identical mutation in the amyloid precursor protein gene (E693delta). *Intern Med* **2015,** 54, (2), 205-8.

18. Shimada, H.; Ataka, S.; Tomiyama, T.; Takechi, H.; Mori, H.; Miki, T., Clinical course of patients with familial early-onset Alzheimer's disease potentially lacking senile plaques bearing the E693Δ mutation in amyloid precursor protein. *Dement Geriatr Cogn Disord* **2011,** 32, (1), 45-54.

19. Shimada, H.; Minatani, S.; Takeuchi, J.; Takeda, A.; Kawabe, J.; Wada, Y.; Mawatari, A.; Watanabe, Y.; Shimada, H.; Higuchi, M.; Suhara, T.; Tomiyama, T.; Itoh, Y., Heavy Tau Burden with Subtle Amyloid β Accumulation in the Cerebral Cortex and Cerebellum in a Case of Familial Alzheimer's Disease with APP Osaka Mutation. *Int J Mol Sci* **2020,** 21, (12).

20. Basun, H.; Bogdanovic, N.; Ingelsson, M.; Almkvist, O.; Näslund, J.; Axelman, K.; Bird, T. D.; Nochlin, D.; Schellenberg, G. D.; Wahlund, L. O.; Lannfelt, L., Clinical and neuropathological features of the arctic APP gene mutation causing early-onset Alzheimer disease. *Arch Neurol* **2008,** 65, (4), 499-505.

21. Kalimo, H.; Lalowski, M.; Bogdanovic, N.; Philipson, O.; Bird, T. D.; Nochlin, D.; Schellenberg, G. D.; Brundin, R.; Olofsson, T.; Soliymani, R.; Baumann, M.; Wirths, O.; Bayer, T. A.; Nilsson, L. N.; Basun, H.; Lannfelt, L.; Ingelsson, M., The Arctic AβPP mutation leads to Alzheimer's disease pathology with highly variable topographic deposition of differentially truncated Aβ. *Acta Neuropathol Commun* **2013,** 1, 60.

22. Lindquist, S. G.; Nielsen, J. E.; Stokholm, J.; Schwartz, M.; Batbayli, M.; Ballegaard, M.; Erdal, J.; Krabbe, K.; Waldemar, G., Atypical early-onset Alzheimer's disease caused by the Iranian APP mutation. *J Neurol Sci* **2008,** 268, (1-2), 124-30.

23. Pasalar, P.; Najmabadi, H.; Noorian, A. R.; Moghimi, B.; Jannati, A.; Soltanzadeh, A.; Krefft, T.; Crook, R.; Hardy, J., An Iranian family with Alzheimer's disease caused by a novel APP mutation (Thr714Ala). *Neurology* **2002,** 58, (10), 1574-5.

24. Lindquist, S. G.; Schwartz, M.; Batbayli, M.; Waldemar, G.; Nielsen, J. E., Genetic testing in familial AD and FTD: mutation and phenotype spectrum in a Danish cohort. *Clin Genet* **2009,** 76, (2), 205-9.

25. Zekanowski, C.; Styczyńska, M.; Pepłońska, B.; Gabryelewicz, T.; Religa, D.; Ilkowski, J.; Kijanowska-Haładyna, B.; Kotapka-Minc, S.; Mikkelsen, S.; Pfeffer, A.; Barczak, A.; Łuczywek, E.; Wasiak, B.; Chodakowska-Zebrowska, M.; Gustaw, K.; Łaczkowski, J.; Sobów, T.; Kuźnicki, J.; Barcikowska, M., Mutations in presenilin 1, presenilin 2 and amyloid precursor protein genes in patients with early-onset Alzheimer's disease in Poland. *Exp Neurol* **2003,** 184, (2), 991-6.

26. Kumar-Singh, S.; De Jonghe, C.; Cruts, M.; Kleinert, R.; Wang, R.; Mercken, M.; De Strooper, B.; Vanderstichele, H.; Löfgren, A.; Vanderhoeven, I.; Backhovens, H.; Vanmechelen, E.; Kroisel, P. M.; Van Broeckhoven, C., Nonfibrillar diffuse amyloid deposition due to a gamma(42)-secretase site mutation points to an essential role for N-truncated A beta(42) in Alzheimer's disease. *Hum Mol Genet* **2000,** 9, (18), 2589-98.

27. Edwards-Lee, T.; Ringman, J. M.; Chung, J.; Werner, J.; Morgan, A.; St George Hyslop, P.; Thompson, P.; Dutton, R.; Mlikotic, A.; Rogaeva, E.; Hardy, J., An African American family with early-onset Alzheimer disease and an APP (T714I) mutation. *Neurology* **2005,** 64, (2), 377-9.

28. Cruts, M.; Dermaut, B.; Rademakers, R.; Van den Broeck, M.; Stögbauer, F.; Van Broeckhoven, C., Novel APP mutation V715A associated with presenile Alzheimer's disease in a German family. *J Neurol* **2003,** 250, (11), 1374-5.

29. Nan, S. J.; Han, Y. Q.; Fan, J.; Chen, Q. H., Blepharospasm in familial AD secondary to an APP mutation (V715M). *Acta Neurol Belg* **2014,** 114, (4), 333-4.

30. Ancolio, K.; Dumanchin, C.; Barelli, H.; Warter, J. M.; Brice, A.; Campion, D.; Frébourg, T.; Checler, F., Unusual phenotypic alteration of beta amyloid precursor protein (betaAPP) maturation by a new Val-715 --> Met betaAPP-770 mutation responsible for probable early-onset Alzheimer's disease. *Proc Natl Acad Sci U S A* **1999,** 96, (7), 4119-24.

31. Park, H. K.; Na, D. L.; Lee, J. H.; Kim, J. W.; Ki, C. S., Identification of PSEN1 and APP gene mutations in Korean patients with early-onset Alzheimer's disease. *J Korean Med Sci* **2008,** 23, (2), 213-7.

32. Guardia-Laguarta, C.; Pera, M.; Clarimón, J.; Molinuevo, J. L.; Sánchez-Valle, R.; Lladó, A.; Coma, M.; Gómez-Isla, T.; Blesa, R.; Ferrer, I.; Lleó, A., Clinical, neuropathologic, and biochemical profile of the amyloid precursor protein I716F mutation. *J Neuropathol Exp Neurol* **2010,** 69, (1), 53-9.

33. Pera, M.; Alcolea, D.; Sánchez-Valle, R.; Guardia-Laguarta, C.; Colom-Cadena, M.; Badiola, N.; Suárez-Calvet, M.; Lladó, A.; Barrera-Ocampo, A. A.; Sepulveda-Falla, D.; Blesa, R.; Molinuevo, J. L.; Clarimón, J.; Ferrer, I.; Gelpi, E.; Lleó, A., Distinct patterns of APP processing in the CNS in autosomal-dominant and sporadic Alzheimer disease. *Acta Neuropathol* **2013,** 125, (2), 201-13.

34. Sieczkowski, E.; Milenkovic, I.; Venkataramani, V.; Giera, R.; Ströbel, T.; Höftberger, R.; Liberski, P. P.; Auff, E.; Wirths, O.; Bayer, T. A.; Kovacs, G. G., I716F AβPP mutation associates with the deposition of oligomeric pyroglutamate amyloid-β and α-synucleinopathy with Lewy bodies. *J Alzheimers Dis* **2015,** 44, (1), 103-14.

35. Blauwendraat, C.; Wilke, C.; Jansen, I. E.; Schulte, C.; Simón-Sánchez, J.; Metzger, F. G.; Bender, B.; Gasser, T.; Maetzler, W.; Rizzu, P.; Heutink, P.; Synofzik, M., Pilot whole-exome sequencing of a German early-onset Alzheimer's disease cohort reveals a substantial frequency of PSEN2 variants. *Neurobiol Aging* **2016,** 37, 208.e11-208.e17.

36. Abstracts from the 8th International Conference on Alzheimer's Disease and Related Disorders. July 20-25, 2002. Stockholm, Sweden. *Neurobiol Aging* **2002,** 23, (1 Suppl), S1-606.

37. Eckman, C. B.; Mehta, N. D.; Crook, R.; Perez-tur, J.; Prihar, G.; Pfeiffer, E.; Graff-Radford, N.; Hinder, P.; Yager, D.; Zenk, B.; Refolo, L. M.; Prada, C. M.; Younkin, S. G.; Hutton, M.; Hardy, J., A new pathogenic mutation in the APP gene (I716V) increases the relative proportion of A beta 42(43). *Hum Mol Genet* **1997,** 6, (12), 2087-9.

38. Zádori, D.; Füvesi, J.; Timár, E.; Horváth, E.; Bencsik, R.; Szépfalusi, N.; Must, A.; Vécsei, L.; Molnár, M. J.; Klivényi, P., The Report of p.Val717Phe Mutation in the APP Gene in a Hungarian Family With Alzheimer Disease: A Phenomenological Study. *Alzheimer Dis Assoc Disord* **2017,** 31, (4), 343-345.

39. Knight, W. D.; Ahsan, R. L.; Jackson, J.; Cipolotti, L.; Warrington, E. K.; Fox, N. C.; Rossor, M. N., Pure progressive amnesia and the APPV717G mutation. *Alzheimer Dis Assoc Disord* **2009,** 23, (4), 410-4.

40. Brooks, W. S.; Martins, R. N.; De Voecht, J.; Nicholson, G. A.; Schofield, P. R.; Kwok, J. B.; Fisher, C.; Yeung, L. U.; Van Broeckhoven, C., A mutation in codon 717 of the amyloid precursor protein gene in an Australian family with Alzheimer's disease. *Neurosci Lett* **1995,** 199, (3), 183-6.

41. Zhang, G.; Xie, Y.; Wang, W.; Feng, X.; Jia, J., Clinical characterization of an APP mutation (V717I) in five Han Chinese families with early-onset Alzheimer's disease. *J Neurol Sci* **2017,** 372, 379-386.

42. Mullan, M.; Tsuji, S.; Miki, T.; Katsuya, T.; Naruse, S.; Kaneko, K.; Shimizu, T.; Kojima, T.; Nakano, I.; Ogihara, T.; et al., Clinical comparison of Alzheimer's disease in pedigrees with the codon 717 Val-->Ile mutation in the amyloid precursor protein gene. *Neurobiol Aging* **1993,** 14, (5), 407-19.

43. Halliday, G.; Brooks, W.; Arthur, H.; Creasey, H.; Broe, G. A., Further evidence for an association between a mutation in the APP gene and Lewy body formation. *Neurosci Lett* **1997,** 227, (1), 49-52.

44. Jiao, B.; Tang, B.; Liu, X.; Xu, J.; Wang, Y.; Zhou, L.; Zhang, F.; Yan, X.; Zhou, Y.; Shen, L., Mutational analysis in early-onset familial Alzheimer's disease in Mainland China. *Neurobiol Aging* **2014,** 35, (8), 1957.e1-6.

45. Lloyd, G. M.; Trejo-Lopez, J. A.; Xia, Y.; McFarland, K. N.; Lincoln, S. J.; Ertekin-Taner, N.; Giasson, B. I.; Yachnis, A. T.; Prokop, S., Prominent amyloid plaque pathology and cerebral amyloid angiopathy in APP V717I (London) carrier - phenotypic variability in autosomal dominant Alzheimer's disease. *Acta Neuropathol Commun* **2020,** 8, (1), 31.

46. Zhou, J.; Chen, Y.; Meng, F.; Zhang, K.; Liu, X.; Peng, G., Presenilin 1 and APP Gene Mutations in Early-Onset AD Families from a Southeast Region of China. *Curr Alzheimer Res* **2020,** 17, (6), 540-546.

47. Ishimaru, T.; Ochi, S.; Matsumoto, T.; Yoshida, T.; Abe, M.; Toyota, Y.; Fukuhara, R.; Tanimukai, S.; Ueno, S., [A case report of early-onset Alzheimer's disease with multiple psychotic symptoms, finally diagnosed as APPV717I mutation by genetic testing]. *Seishin Shinkeigaku Zasshi* **2013,** 115, (10), 1042-50.

48. Matsumura, Y.; Kitamura, E.; Miyoshi, K.; Yamamoto, Y.; Furuyama, J.; Sugihara, T., Japanese siblings with missense mutation (717Val --> Ile) in amyloid precursor protein of early-onset Alzheimer's disease. *Neurology* **1996,** 46, (6), 1721-3.

49. Godbolt, A. K.; Beck, J. A.; Collinge, J. C.; Cipolotti, L.; Fox, N. C.; Rossor, M. N., A second family with familial AD and the V717L APP mutation has a later age at onset. *Neurology* **2006,** 66, (4), 611-2.

50. Murrell, J. R.; Hake, A. M.; Quaid, K. A.; Farlow, M. R.; Ghetti, B., Early-onset Alzheimer disease caused by a new mutation (V717L) in the amyloid precursor protein gene. *Arch Neurol* **2000,** 57, (6), 885-7.

51. Abe, M.; Sonobe, N.; Fukuhara, R.; Mori, Y.; Ochi, S.; Matsumoto, T.; Mori, T.; Tanimukai, S.; Ueno, S., Phenotypical difference of amyloid precursor protein (APP) V717L mutation in Japanese family. *BMC Neurol* **2012,** 12, 38.

52. Ghidoni, R.; Albertini, V.; Squitti, R.; Paterlini, A.; Bruno, A.; Bernardini, S.; Cassetta, E.; Rossini, P. M.; Squitieri, F.; Benussi, L.; Binetti, G., Novel T719P AbetaPP mutation unbalances the relative proportion of amyloid-beta peptides. *J Alzheimers Dis* **2009,** 18, (2), 295-303.

53. Wang, Q.; Jia, J.; Qin, W.; Wu, L.; Li, D.; Wang, Q.; Li, H., A Novel AβPP M722K Mutation Affects Amyloid-β Secretion and Tau Phosphorylation and May Cause Early-Onset Familial Alzheimer's Disease in Chinese Individuals. *J Alzheimers Dis* **2015,** 47, (1), 157-65.

54. Kwok, J. B.; Li, Q. X.; Hallupp, M.; Whyte, S.; Ames, D.; Beyreuther, K.; Masters, C. L.; Schofield, P. R., Novel Leu723Pro amyloid precursor protein mutation increases amyloid beta42(43) peptide levels and induces apoptosis. *Ann Neurol* **2000,** 47, (2), 249-53.

55. Theuns, J.; Marjaux, E.; Vandenbulcke, M.; Van Laere, K.; Kumar-Singh, S.; Bormans, G.; Brouwers, N.; Van den Broeck, M.; Vennekens, K.; Corsmit, E.; Cruts, M.; De Strooper, B.; Van Broeckhoven, C.; Vandenberghe, R., Alzheimer dementia caused by a novel mutation located in the APP C-terminal intracytosolic fragment. *Hum Mutat* **2006,** 27, (9), 888-96.

56. Nilsberth, C.; Westlind-Danielsson, A.; Eckman, C. B.; Condron, M. M.; Axelman, K.; Forsell, C.; Stenh, C.; Luthman, J.; Teplow, D. B.; Younkin, S. G.; Näslund, J.; Lannfelt, L., The 'Arctic' APP mutation (E693G) causes Alzheimer's disease by enhanced Abeta protofibril formation. *Nat Neurosci* **2001,** 4, (9), 887-93.

57. Lin, Y. C.; Wang, J. Y.; Wang, K. C.; Liao, J. Y.; Cheng, I. H., Differential regulation of amyloid precursor protein sorting with pathological mutations results in a distinct effect on amyloid-β production. *J Neurochem* **2014,** 131, (4), 407-12.

58. Li, N. M.; Liu, K. F.; Qiu, Y. J.; Zhang, H. H.; Nakanishi, H.; Qing, H., Mutations of beta-amyloid precursor protein alter the consequence of Alzheimer's disease pathogenesis. *Neural Regen Res* **2019,** 14, (4), 658-665.

59. De Jonghe, C.; Esselens, C.; Kumar-Singh, S.; Craessaerts, K.; Serneels, S.; Checler, F.; Annaert, W.; Van Broeckhoven, C.; De Strooper, B., Pathogenic APP mutations near the gamma-secretase cleavage site differentially affect Abeta secretion and APP C-terminal fragment stability. *Hum Mol Genet* **2001,** 10, (16), 1665-71.

60. Suárez-Calvet, M.; Belbin, O.; Pera, M.; Badiola, N.; Magrané, J.; Guardia-Laguarta, C.; Muñoz, L.; Colom-Cadena, M.; Clarimón, J.; Lleó, A., Autosomal-dominant Alzheimer's disease mutations at the same codon of amyloid precursor protein differentially alter Aβ production. *J Neurochem* **2014,** 128, (2), 330-9.

61. Tamaoka, A.; Odaka, A.; Ishibashi, Y.; Usami, M.; Sahara, N.; Suzuki, N.; Nukina, N.; Mizusawa, H.; Shoji, S.; Kanazawa, I.; et al., APP717 missense mutation affects the ratio of amyloid beta protein species (A beta 1-42/43 and a beta 1-40) in familial Alzheimer's disease brain. *J Biol Chem* **1994,** 269, (52), 32721-4.

62. Hsu, S.; Gordon, B. A.; Hornbeck, R.; Norton, J. B.; Levitch, D.; Louden, A.; Ziegemeier, E.; Laforce, R., Jr.; Chhatwal, J.; Day, G. S.; McDade, E.; Morris, J. C.; Fagan, A. M.; Benzinger, T. L. S.; Goate, A. M.; Cruchaga, C.; Bateman, R. J.; Karch, C. M., Discovery and validation of autosomal dominant Alzheimer's disease mutations. *Alzheimers Res Ther* **2018,** 10, (1), 67.
